# Case report: a rare case of primary hyperparathyroidism due to an intrathymic ectopic parathyroid adenoma incidentally diagnosed in a 15-year-old girl

**DOI:** 10.3389/fendo.2024.1371098

**Published:** 2024-10-08

**Authors:** Ercument Gurluler

**Affiliations:** Department of General Surgery, Uludag University Faculty of Medicine, Bursa, Türkiye

**Keywords:** ectopic parathyroid adenoma, pediatric age, persistent hypercalcemia, persistently elevated PTH, VATS thymectomy, combined MIBI plus CT, intraoperative intact PTH monitoring, frozen section diagnosis

## Abstract

Primary hyperparathyroidism (PHPT) due to ectopic parathyroid adenoma is a rare case of hypercalcemia in the pediatric population. Herein, a rare case of PHPT due to ectopic intrathymic parathyroid adenoma was described in an asymptomatic 15-year-old girl who had incidental diagnosis based on laboratory abnormalities but experienced a 3-month postoperative course of persistently elevated parathyroid hormone (PTH) and hypercalcemia following the initial unsuccessful parathyroidectomy operation carried out in a non-parathyroid expert center. The curative surgical treatment was accomplished only after the patient was reoperated with video-assisted thoracoscopic surgery (VATS) thymectomy by the surgeon experienced in parathyroid surgery with implementation of the combined imaging modalities for accurate localization of ectopic adenoma including 99mTc sestamibi (MIBI) plus neck and thoracic computed tomography (CT) and the appropriate surgical strategies including intraoperative intact PTH monitoring and frozen section diagnosis. Before the reoperation (VATS thymectomy), laboratory findings showed elevated PTH (1,171 ng/L; reference range: 21.80 ng/L–87.5 ng/L) and hypercalcemia (13.4 mg/dL; reference range: 8.4 mg/dL–10.2 mg/dL). The preoperative PTH levels were 94 ng/L at 5 min after thymectomy and 78 ng/L at 10 min. The PTH and calcium levels were 54.3 ng/L and 8.47 mg/dL, respectively, on postoperative day 1 and were 34.2 ng/L and 8.1 mg/dL on postoperative day 2. The patient was discharged on postoperative day 2 without any complications. In conclusion, our findings indicate the likelihood of isolated primary hyperparathyroidism to be incidentally diagnosed based solely on laboratory abnormalities with no specific clinical manifestations in the pediatric age. In addition, using combined imaging modalities (such as MIBI and CT) in accurate localization of ectopic parathyroid adenoma and implementation of surgery by experienced surgeons along with intraoperative intact PTH monitoring and frozen section diagnosis seem crucial to ensure the curative surgical treatment.

## Introduction

Primary hyperparathyroidism (PHPT) is not a common diagnosis in the pediatric population with an estimated incidence of 1 per 200–300,000 and is principally caused by a single parathyroid adenoma in this age group (1, 2). PHPT is a rare case of hypercalcemia in children, accounting for <5% of the cases, which typically presents with characteristic signs/symptoms or may be diagnosed incidentally in a small subset of patients (1, 3).

Ectopic parathyroid adenomas, which may be located anywhere in the trajectory from the tongue to the mediastinum, are also rare in children comprising 5% to 26% of total parathyroid adenomas, whereas the most common ectopic location is considered to be the thymus (1, 2, 4, 5).

In all pediatric PHPT patients, surgical resection of the parathyroid gland is the only curative option, and the accurate preoperative localization of parathyroid adenomas is essential to the success of minimally invasive parathyroidectomy (6, 7).

For ectopic parathyroid glands located in either the anterior or posterior mediastinum, the use of minimally invasive surgical techniques such as video-assisted thoracoscopic surgery (VATS) and video-assisted mediastinoscopy (VAM) has been reported to achieve surgical outcomes similar to those associated with the traditional surgical approach (median sternotomy or thoracotomy), in addition to the advantages of short hospital stays and cost (8–10). However, despite being utilized in adult patients for decades, the use of a thoracoscopic approach has been reported only in a few recent pediatric cases (7, 11–13).

Although the prognosis is usually favorable without complications after curative parathyroid resection, emerging data suggest that a substantial number of patients continue to have elevated parathyroid hormone (PTH) levels post-parathyroidectomy with nearly half demonstrating elevated PTH at some point during follow-up (14–17). Persistent hyperparathyroidism after initial surgery is considered to be mainly due to failure to remove an overactive parathyroid (i.e., adenoma or unrecognized parathyroid hyperplasia) (17–19).

Herein, we present a rare case of PHPT due to ectopic intrathymic parathyroid adenoma in a 15-year-old girl, which was incidentally diagnosed based on laboratory abnormalities and successfully managed only after the reoperation with VATS thymectomy in experienced hands after a 3-month postoperative course of persistently elevated PTH and hypercalcemia and continued presence of thymic adenoma following the initial unsuccessful parathyroidectomy.

## Case presentation

**Figure 1:**
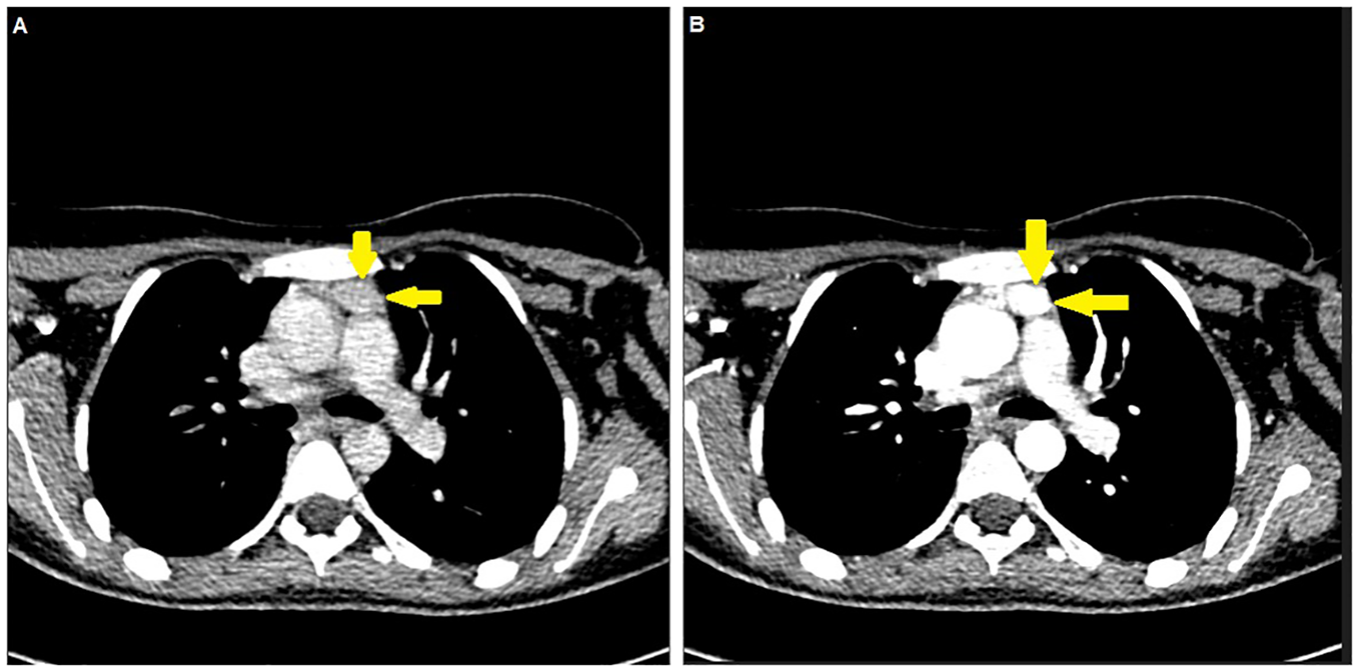
An ectopic thymic parathyroid adenoma detected by **(A)** neck (venous phase) computed tomography and **(B)** thoracic (arterial phase) computed tomography (yellow arrows).

**Figure 2:**
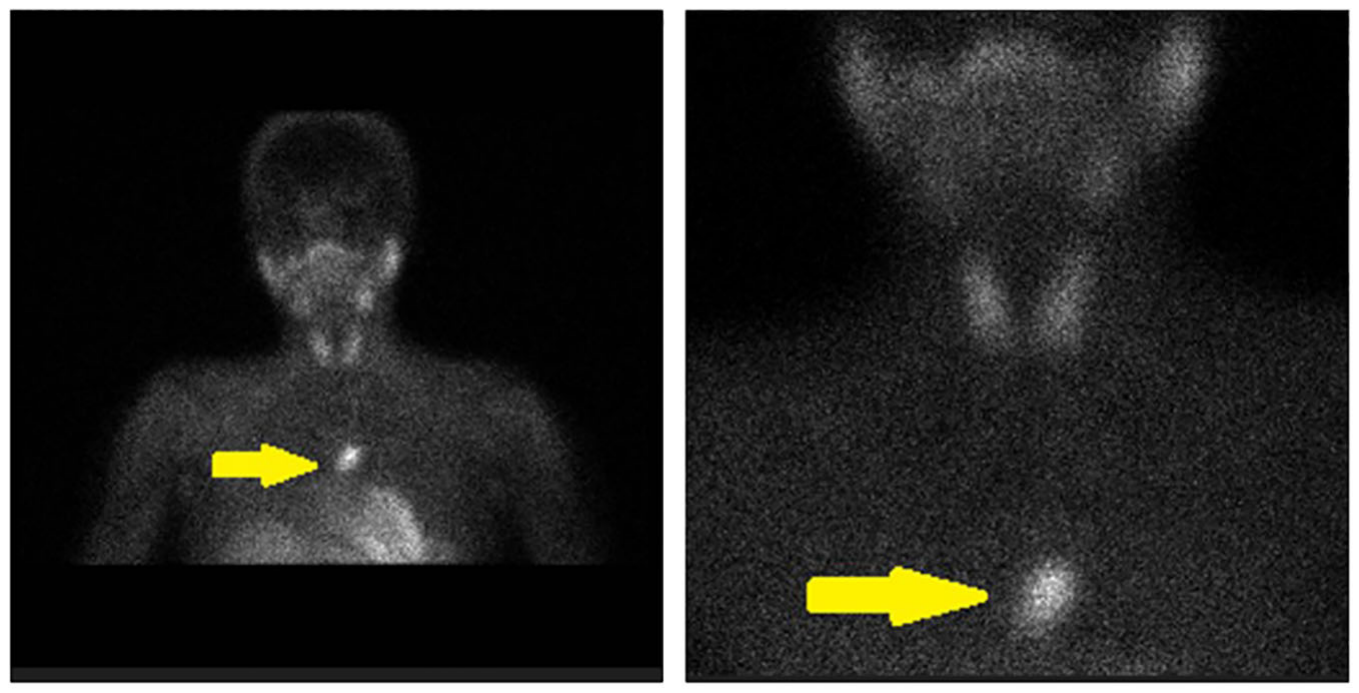
An ectopic thymic parathyroid adenoma detected by 99mTc-sestamibi (MIBI) scanning (yellow arrows).

**Figure 3:**
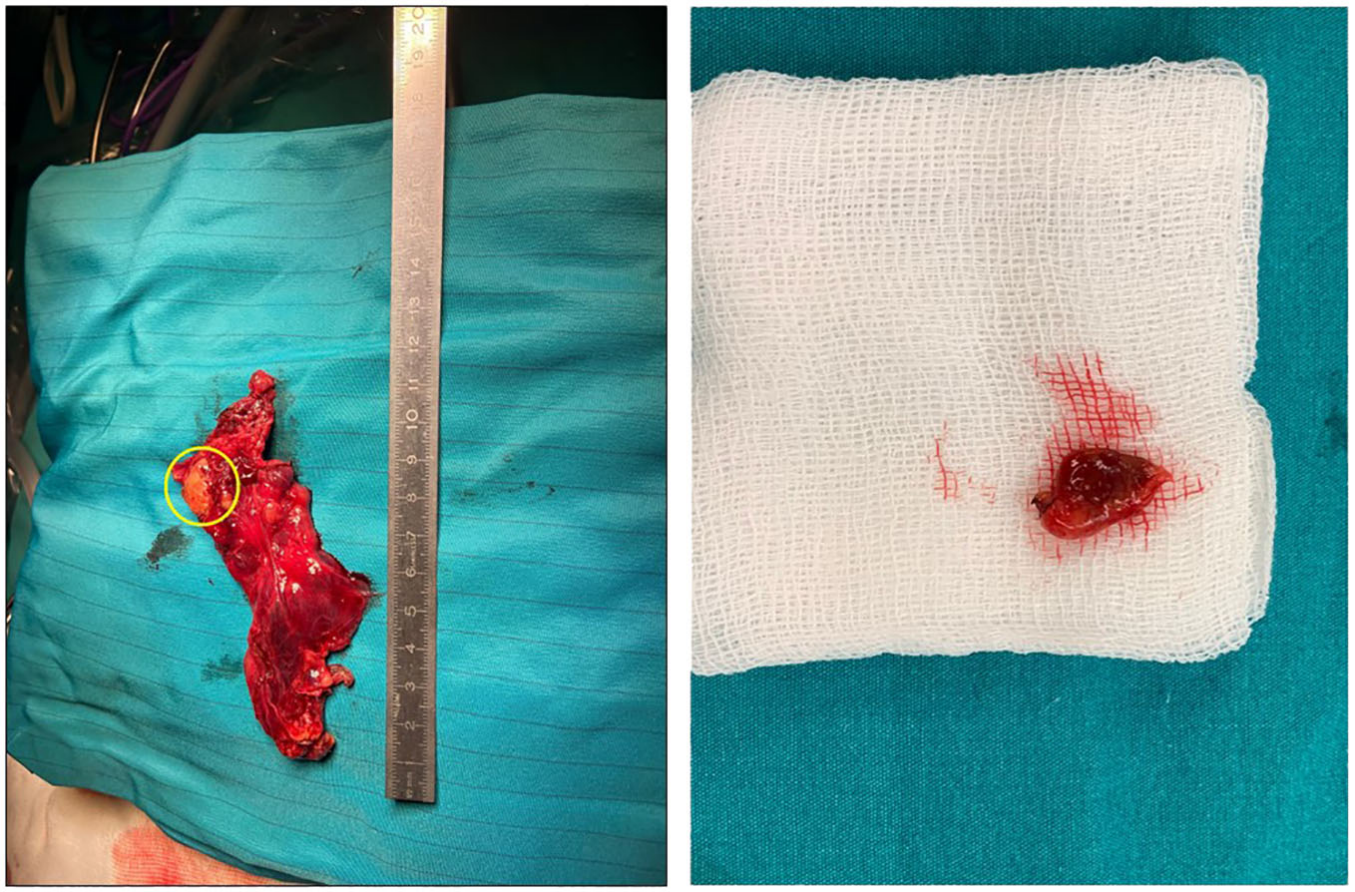
The gross appearance of an ectopic parathyroid adenoma. A 2.7 cm well-demarcated mass located within the thymus (yellow circle).

A 15-year-old girl was referred to our general surgery clinic with postoperative hypercalcemia and elevated PTH levels persisting for 3 months since the thoracoscopic intrathoracic left parathyroidectomy operation for PHPT carried out in another center. The anamnesis revealed incidental detection of hypercalcemia and elevated PTH levels during her previous hospitalization with grade 3 splenic laceration after intra-vehicle traffic accident (on July 2023). During the pediatric endocrinology consultation for further investigation of hypercalcemia and elevated PTH levels (August 2023), the laboratory investigation revealed hypercalcemia (15.1 mg/dL) and elevated PTH levels (1,685.8 ng/L), in addition to serum phosphorus (4.2 mg/dL), ALP (191 U/L), vitamin D (6.6 µg/L), and spot urinalysis (albumin/creatinine: 66 mg/g; microalbumin: 2.4 mg/dL; phosphorus: 49.27 mg/dL; calcium: 22.20 mg/dL; creatinine: 36.98 mg/dL; urea: 965.50 mg/dL) findings. The neck ultrasound revealed normal findings for the thyroid gland with no nodular lesion with clear boundaries in the thyroid or space-occupying lesions with clear borders in the parathyroid lodge, and no ultrasonographical pathological lymph nodes in the bilateral cervical lymphatic chain. Technetium 99m sestamibi (MIBI) for the neck and thorax showed a focus (25 × 15 mm) of increased activity in the anterior mediastinum at a 70-mm distance to the left thyroid lobe in early-phase planar images, and persistence of the focal increased activity in the delayed-phase images, consistent with ectopic mediastinal parathyroid adenoma. Upon these findings, the patient underwent thoracoscopic intrathoracic left parathyroidectomy (in August 2023) with suspected diagnosis of ectopic parathyroid adenoma but intraoperative intact PTH monitoring, and frozen section diagnosis were not applied during the surgery. The pathology of resected material indicated the benign lymph node. Hence, the serum calcium and PTH levels remained to be elevated postoperatively and the pathological diagnosis was consistent with benign lymph nodules. Upon persistence of hypercalcemia and PTH elevation during the 3-month postoperative follow-up, the patient was referred to our clinic on November 2023. In the current physical examination, height was 148.5 cm (percentile: 1.1, age for height: 11.4 years), weight was 45.7 kg (percentile 5.05, age for height: 12.12 years), and body mass index (BMI) was found to be 20.72 kg/m^2^ (percentile: 44.43). The laboratory findings in the current examination showed elevated PTH (1,171 ng/L; reference range: 21.80 ng/L–87.5 ng/L), hypercalcemia (13.4 mg/dL; reference range: 8.4 mg/dL–10.2 mg/dL), normal albumin (4.3 g/L; reference range: 3.8 g/dL–5.4 g/dL), hypophosphatemia (3.2 mg/dL; reference range, 3.8 mg/dL–5.9 mg/dL), elevated ALP (302 U/L; reference range: 50 U/L–162 U/L), and vitamin D deficiency (25-OH-vitamin D level of 6.6 ng/mL; reference range: 30 ng/mL–100 ng/mL). Results of the 24-h uranalysis revealed daily calcium excretion of 600 mg and uric acid excretion of 600 mg. The diagnostic investigation for MEN 1 syndrome revealed negative findings The diagnostic investigation for MEN 1 syndrome revealed negative findings, including the pituitary MRI (absence of pituitary adenoma), abdominal CT (no pancreatic or adrenal pathology), and MEN1 gene mutational analysis (negative for the causative oncosuppressor gene MEN1 at the 11q13 region). MIBI scanning and single-photon emission CT (SPECT/CT) revealed the persistence of an ectopic thymic parathyroid adenoma (**Figures 1**, **2**). Accordingly, the patient underwent the right video-assisted thoracoscopic surgery (VATS) thymectomy in December 2023 by the expert parathyroid surgeon following the intraoperative intact PTH monitoring and frozen section diagnosis protocols. The preoperative PTH levels were 94 ng/L at 5 min after thymectomy and 78 ng/L at 10 min. Sectioning of the specimen was performed, and it showed a 2.7-cm well-demarcated intrathymic mass (**Figure 3**). Pathological findings confirmed an intrathymic ectopic parathyroid adenoma. The PTH and calcium levels were 54.3 ng/L and 8.47 mg/dL, respectively, on postoperative day 1 and were 34.2 ng/L and 8.1 mg/dL on postoperative day 2. The vitamin D level was 10.0 µg/L, and the ALP level was 186 U/L on postoperative day 2. The patient was discharged on postoperative day 2 without any complications. The PTH and calcium levels remained within the normal range during the 6-month postoperative monitoring.

## Discussion

Herein, a rare case of incidentally diagnosed ectopic thymic parathyroid adenoma was described in a 15-year-old girl who had persistent hypercalcemia and elevated PTH for 3 months after the initial presumed curative parathyroidectomy in another center and reoperated in experienced hands with VATS thymectomy, which revealed normalization of PTH and calcium levels early in the postoperative period. Our findings emphasize the crucial role of using combined imaging modalities (such as MIBI and CT) in accurate localization of ectopic parathyroid adenoma in the pediatric age and implementation of the surgery by experienced surgeons with concomitant intraoperative intact PTH monitoring and frozen section diagnosis to ensure the curative surgical treatment.

Likewise, the use of combined imaging modalities (MIBI plus CT) is considered of critical importance in identifying the ectopic location in pediatric parathyroid adenoma cases, which otherwise delays the implementation of the definitive surgical treatment (7, 11, 14, 20). In addition to the use of appropriate imaging modalities in preoperative detection of the adenoma, implementing appropriate intraoperative strategies (intraoperative intact PTH monitoring and frozen section diagnosis) by surgeons experienced in parathyroid surgery is considered imperative for successful management of disease (11, 20).

In contrast to adult patients, PHPT is considered to be symptomatic in the pediatric age with almost 80% of pediatric patients presenting with symptoms of hypercalcemia and target organ (bone and renal) involvement rather than abnormal laboratory values alone (13, 14). However, in our case, the isolated PHPT was incidentally diagnosed in an asymptomatic child on the basis of laboratory abnormalities (hypercalcemia, elevated PTH, and hypophosphatemia). Similarly, the likelihood of sporadic isolated PHPT to manifest with asymptomatic hypercalcemia and an ectopic location, and to be diagnosed based solely on laboratory abnormalities with no specific presenting signs or symptoms, was also noted in previous pediatric case reports (7, 11, 12, 20).

Parathyroid glands are frequently ectopic due to their complex migration during embryological development, and the ectopic glands are frequently found in the mediastinum, in an intrathymic rather than in a parathymic location (11, 21). Similar to our case, the previous studies on ectopic parathyroid adenomas in pediatric patients also revealed the localization of the adenoma to be associated with the thymus approximately in half of the cases, suggesting that this region should be carefully evaluated in imaging-based search to locate ectopic parathyroid adenomas in children (5, 7, 12).

All patients with PHPT in the pediatric age group require surgical resection of the parathyroid glands for definitive treatment, necessitating the preoperative parathyroid imaging to localize an ectopic parathyroid (7, 22). However, there is no consensus for the optimal imaging modality in identifying ectopic parathyroid adenomas in the pediatric population, which is considered a challenging process with use of at least two different imaging modalities in most patients preoperatively (11, 12). Use of neck ultrasound is considered ineffective in localizing ectopic parathyroid adenomas, whereas MIBI is suggested to have a lower sensitivity in children (10%) than in adults (66%–81%) (2, 11, 21, 23). Nonetheless, use of combined modalities such as MIBI, single-photon emission CT (SPECT/CT), and positron emission tomography/CT have been increasingly reported in the literature in terms of their favorable utility in verifying the ectopic location of an adenoma, predicting the location of adenoma within the thymus, and guiding the surgical localization, especially for minimally invasive procedures (2, 4, 12, 24, 25). MIBI and SPECT/CT have become the most commonly reported imaging modality in pediatric ectopic parathyroid adenomas (2, 12). Although 4D CT has been increasingly used in reoperations, especially for the difficult undiagnosed-by-MIBI and hyperplasia cases, its use in pediatric cases may be limited due to high radiation exposure (2, 12, 17, 26).

The anatomical variations related to the mediastinal located parathyroid adenoma on preoperative imaging are well known to parathyroid surgeons, which usually indicates the presence of a large number of parathyroid glands (8, 23, 27). Hence, while the success rate of surgical treatment is more than 95% in experienced hands, the ectopic site of responsible parathyroid gland, inadequate experience of the surgeon, insufficient cooperation with pathologist, and failure to recognize histopathological lesion are considered among the main causes of operative treatment failure for PHPT (7, 17, 28, 29). Likewise, the poor imaging and surgical strategies followed in a non-parathyroid expert center led to reoperation decision in our patient, due to persistent hypercalcemia, and elevated PTH levels in the presence of thymic adenoma failed to be removed during the initial operation which was performed with use of neither the intraoperative PTH monitoring nor the frozen section diagnosis along with the final pathological diagnosis of benign lymph nodes.

The second operation carried out in our center was based on VATS thymectomy along with intraoperative PTH monitoring and frozen section diagnosis. Hence, our findings support that with proper patient selection, appropriate and adequate preoperative imaging for adenoma localization, and intraoperative PTH monitoring, a thoracoscopic parathyroidectomy is safe and feasible option in the surgical management of PHPT secondary to an ectopic mediastinal parathyroid adenoma in pediatric patients (7, 11, 12, 23, 30, 31).

Moreover, our findings indicate that the extensive thymectomy is also eligible via VATS, which seems notable given that it is considered more productive to identify and remove the thymus than to seek blindly in the mediastinum in search of the adenoma, when parathyroids are not found in their usual positions (8, 23).

In general, high preoperative PTH, larger adenomas, and vitamin D deficiency are considered to be the risk factors for persistently elevated PTH after parathyroidectomy (4, 12, 15–17, 32). In this regard, the markedly high preoperative PTH levels and persistent vitamin D deficiency in our case should also be considered in terms of their further contribution to persistent hyperparathyroidism caused by the failed surgery. Nonetheless, the decline in PTH levels after VAS thymectomy was also accompanied by an increase in the vitamin D levels (from 6 µg/L to 10.0 µg/L) even in the early postoperative period in our patient, which are indicators of good outcome minimizing the likelihood of future persistence (17).

## Conclusion

In conclusion, our findings indicate the likelihood of isolated primary hyperparathyroidism to be incidentally diagnosed based solely on laboratory abnormalities with no specific clinical manifestations in the pediatric age. In addition, using combined imaging modalities (such as MIBI and CT) in accurate localization of ectopic parathyroid adenoma and implementation of surgery by experienced surgeons along with intraoperative intact PTH monitoring and frozen section diagnosis seems crucial to ensure the curative surgical treatment.

## Data Availability

The original contributions presented in the study are included in the article/supplementary material. Further inquiries can be directed to the corresponding author.
